# “When physical change transforms the mind: bariatric surgery and bipolar disorder.” A case study

**DOI:** 10.1192/j.eurpsy.2026.11197

**Published:** 2026-07-31

**Authors:** M. I. Pinedo De Oro, L. A. Gutierrez, P. Barcelo, K. T. Oyola, S. Perneth

**Affiliations:** Atlantico, Universidad Simon Bolivar, Barranquilla, Colombia

## Abstract

**Introduction:**

According to the WHO, obesity is a chronic disease characterized by excessive fat accumulation, representing a risk factor for various physical and mental disorders. Worldwide, 890 million people are obese, and in Colombia, its prevalence is 18.7%. Due to low adherence to conventional treatments, bariatric surgery has become established as an effective and lasting weight-loss option. While the physical benefits of bariatric surgery are well-documented, its psychological effects are not yet fully understood. Some studies report an increased incidence of mood disorders. Bipolar disorder, characterized by mood swings, has also been described in some postoperative cases. Our objective is to describe a clinical case where bariatric surgery is identified as a possible trigger for mental illness.

**Objectives:**

to describe a case of bipolar disorder emerging after bariatric surgery and emphasize the need for psychiatric follow- up to prevent postoperative mood complications

**Methods:**

A descriptive single-case study was conducted through a retrospective review of medical records, structured psychiatric interviews, and standardized psychometric assessments: Hamilton Depression Rating Scale (HDRS) for depressive symptoms and Young Mania Rating Scale (YMRS) for hypomanic symptoms.

**Results:**

A 34-year-old woman with a history of obesity and compensatory eating behaviors since adolescence underwent gastric bypass surgery at age 26. In the following months, she experienced an untreated postpartum depressive episode, followed by mood fluctuations characterized by hypomanic periods with increased energy, impulsivity, decreased need for sleep, and hypersexuality, alternating with depressive episodes marked by anhedonia, low motivation, and generalized insomnia. During her most recent episode, she presented with depressive symptoms consistent with Bipolar II Disorder; however, during hospitalization, she developed hypomanic symptoms and showed a favorable response to quetiapine as a mood stabilizer.

**Image 1:**
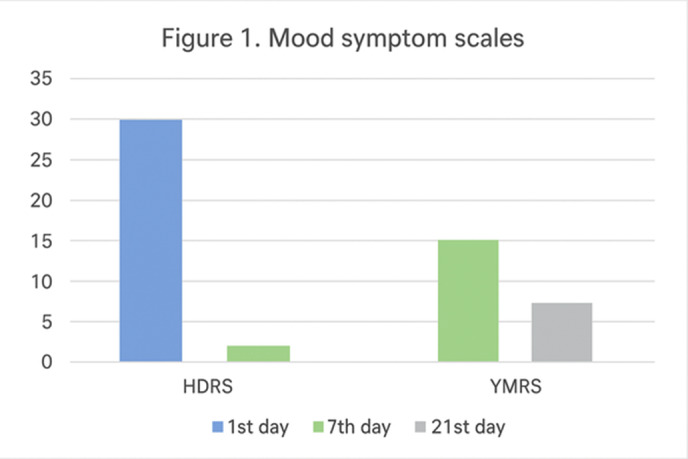

**Conclusions:**

This case highlights a possible association between bariatric surgery and the emergence of Bipolar II Disorder in a patient without prior major psychiatric history. Physiological and psychosocial changes after the procedure may act as triggers for affective dysregulation. The use of standardized psychometric scales facilitated the objective assessment of symptom severity and diagnostic clarification.

**Disclosure of Interest:**

None Declared

